# IGSF11 and VISTA: a pair of promising immune checkpoints in tumor immunotherapy

**DOI:** 10.1186/s40364-022-00394-0

**Published:** 2022-07-13

**Authors:** Xi-Yang Tang, Yan-Lu Xiong, Xian-Gui Shi, Ya-Bo Zhao, An-Ping Shi, Kai-Fu Zheng, Yu-Jian Liu, Tao Jiang, Nan Ma, Jin-Bo Zhao

**Affiliations:** 1 Department of Thoracic Surgery, Tangdu Hospital, Air Force Medical University, 569 Xinsi Road, Xi’an, 710038 Shaanxi China; 2 College of Basic Medicine, Air Force Medical University, Xi’an, 710032 Shaanxi China; 3 Department of Radiology & Functional and Molecular Imaging Key Lab of Shaanxi Province, Tangdu Hospital, Fourth Military Medical University (Air Force Medical University), 569 Xinsi Road, Xi’an, 710038 Shaanxi China; 4 Department of Ophthalmology, Tangdu Hospital, Air Force Medical University, 569 Xinsi Road, 710038 Xi’an, China

**Keywords:** IGSF11, VISTA, Tumor, Immune checkpoint, Immunotherapy

## Abstract

Immunotherapy has become the major treatment for tumors in clinical practice, but some intractable problems such as the low response rate and high rates of immune-related adverse events still hinder the progress of tumor immunotherapy. Hence, it is essential to explore additional immunotherapy treatment targets. In this review, we focus on the structure, expression and expression-related mechanisms, interactions, biological functions and the progress in preclinical/clinical research of IGSF11 and VISTA in tumors. We cover the progress in recent research with this pair of immune checkpoints in tumor immune regulation, proliferation, immune resistance and predictive prognosis. Both IGSF11 and VISTA are highly expressed in tumors and are modulated by various factors. They co-participate in the functional regulation of immune cells and the inhibition of cytokine production. Besides, in the downregulation of IGSF11 and VISTA, both inhibit the growth of some tumors. Preclinical and clinical trials all emphasize the predictive role of IGSF11 and VISTA in the prognosis of tumors, and that the predictive role of the same gene varies from tumor to tumor. At present, further research is proving the enormous potential of IGSF11 and VISTA in tumors, and especially the role of VISTA in tumor immune resistance. This may prove to be a breakthrough to solve the current clinical immune resistance, and most importantly, since research has focused on VISTA but less on IGSF11, IGSF11 may be the next candidate for tumor immunotherapy.

**Supplementary Information:**

The online version contains supplementary material available at 10.1186/s40364-022-00394-0.

## Introduction

**Fig. 1:**
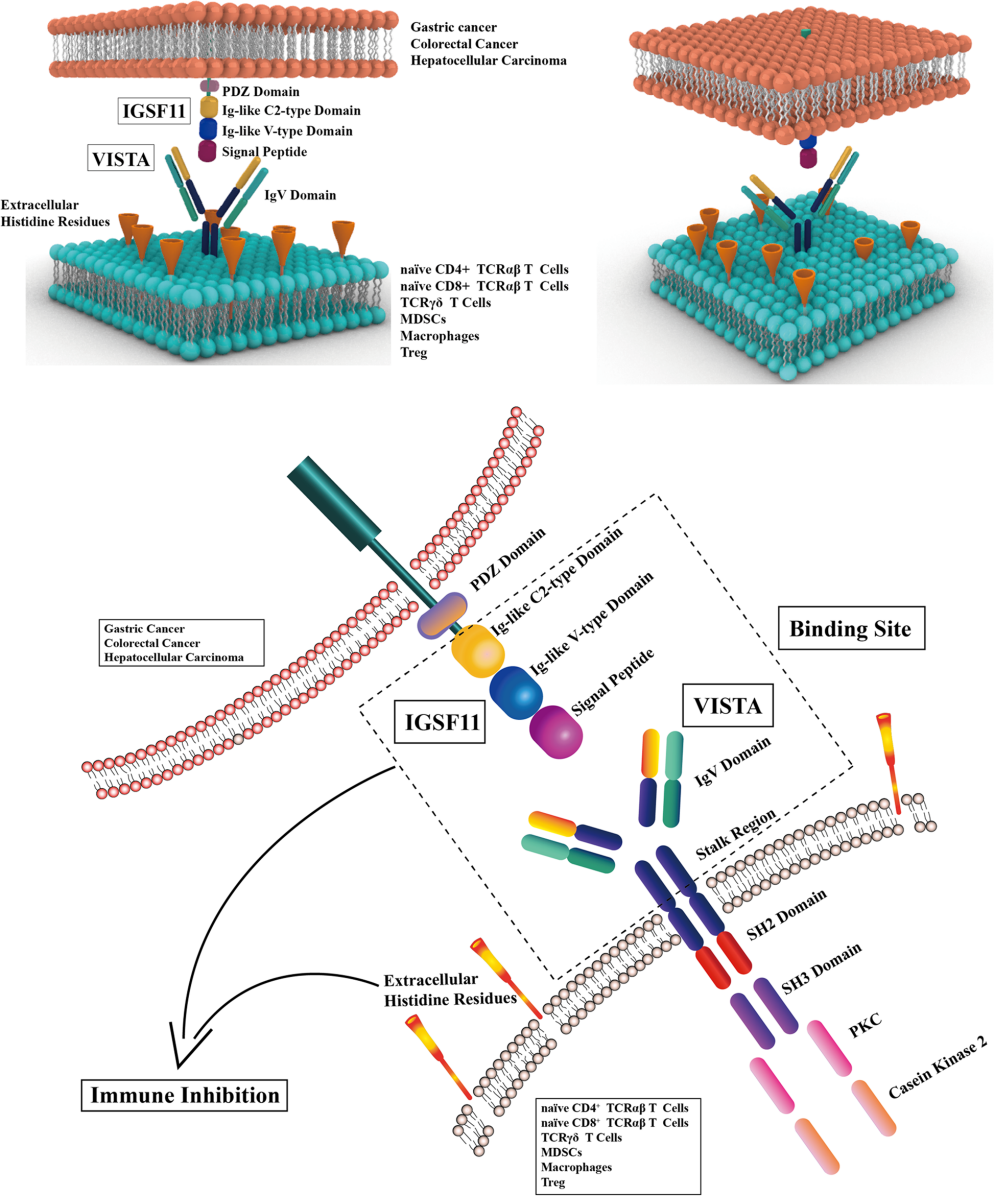
[The structure, expression, binding site and the immune regulation of IGSF11 and VISTA]. (a) Pattern diagram of the interaction between IGSF11 and VISTA. IGSF11 binds with VISTA mainly by V-type and C-type immunoglobulin-like domain. (b) The membrane expression, binding site and domains related with immune regulation of IGSF11 and VISTA

Immunotherapy has become the major therapeutic method for tumors with a favorable treatment effect. Various immune checkpoints are the mature targets in tumor immunotherapy, like PD1/PD-L1 (Programmed Cell Death 1/ Programmed Cell Death Ligand 1), CTLA4 (Cytotoxic T-Lymphocyte Associated Protein 4) and LAG3 (Lymphocyte Activating 3), are in ongoing clinical trials [1]. But there are still some intractable problems, since the response rate of immunotherapy is too low but the irAEs (immune-related adverse events) are relatively high. For example, the response rate of anti-PD1/PD-L1 monotherapy or combination therapy is only about 30% in non-small cell lung cancer (NSCLC) [2], but the irAEs’ incidence of anti-CTLA-4 inhibitors is nearly 70% in 1265 oncologic patients from 22 clinical trials [3]. Thus, it is urgent to explore novel immune checkpoints with a higher response rate and lower incidence of irAEs, and we focus on IGSF11 and VISTA. IGSF11 (immunoglobulin superfamily 11 gene, also known as BT-IgSF, BTIGSF, CT119, CXADRL1, VSIG3) belongs to the immunoglobulin superfamily, and is a 46 KDa protein containing 431 amino acids. It is located on chromosome 3q13.32, exerts in cell adhesion [4, 5], migration [6], proliferation, differentiation [4, 7, 8], synapses’ induction [9, 10], maintaining the integrity of blood-testis barrier [5, 11] and meiotic diplotene of somatic cells and germ cells [12]. Besides this, it serves as the ligand of VISTA, regulating the function of immune cells, especially for T cells [13]. VISTA (V-domain immunoglobulin suppressor of T cell activation, also known as VSIR, B7-H5, B7H5, C10orf54, Dies1, PD-1H, SISP1), belongs to the immunoglobulin family, but is limited with other B7 family members, VISTA is a 34 KDa protein containing 311 amino acids, located on chromosome 10q22.1. There are two proved ligands of VISTA, one is IGSF11, the other is PSGL1 (P-selectin glycoprotein ligand 1) [13, 14]. Besides these, VSIG8, NSC622608 and Galectin 9 may be potential ligands for VISTA [15, 16]. The interaction between ligands and receptors may be modulated by the pH in microenvironment [14], and exert innate and adaptive immune regulation [17, 18]. The overexpression of VISTA may induce the secretion of TNF-α, IL-10, IL-6 and IL-1B [19], whereas the deficiency of VISTA may affect the production of MIG, IP10, MCP-1, the number of CD4^+^ T cells in blood and myeloid cells in spleen [20, 21]. Besides which, VISTA can also function in microglia inflammation [22, 23], and chemokines’ responsiveness [24]. IGSF11 and VISTA are also a pair of immune checkpoints that exert in tumor proliferation and immune regulation [25], which has enormous potential to be used as a novel tumor immunotherapy target and biomarker [26]. In this review, we illuminate the structure, expression, biological effect and clinical application of IGSF11 and VISTA, aiming at summarizing the recent research progress for further exploration (Fig. 1).

## IGSF11/VISTA structure and expression

### IGSF11/VISTA structure

The structural research about IGSF11 and VISTA may provide more targets for specific antibody design. IGSF11 is a type I transmembrane protein containing an extracellular domain, transmembrane domain and cytoplasmic domain [13]. Three domains construct the extracellular domain, including the C-type domain, V-type domain (with signal peptide) and PDZ domain; the V-type and C-type immunoglobulin-like domain are responsible for binding with VISTA [13]. The crystal structure of the extracellular domain is identified at 2.64 angstrom resolution, which may provide the structural basis for IGSF11 antibody [27]. The structure of VISTA contains as follows: a large 130 aa IgV-like domain linked with a 33 aa stalk segment; a 20 aa transmembrane region linked with a 96 aa cytoplasmic tail [28, 29]. The concentration of histidine residues in the extracellular domain is striking, and is involved in the inhibition of T cell activation [30]; SH2 domain (Src homology domain 2) locates at the middle of the cytoplasmic tail, SH3 domain (Src homology domain 3) is also a part of VISTA, casein kinase 2 and phosphokinase C phosphorylation sites consisting of the cytoplasmic domain [15, 31]. The crystal structure of VISTA is unique because of its extended CC’ loop region (a target of the VISTA block) with an attached helix and two disulfide bonds, the binding epitope of VISTA overlaps with IGSF11, and research on the VISTA crystal structure may also provide a novel target for antibody design [29].

### IGSF11/VISTA expression and expression-related regulation mechanisms

Both IGSF11 and VISTA are highly expressed in tumors, the expression and expression-related regulation mechanisms of IGSF11 and VISTA are listed in Table 1. Compared to the minimal expression of IGSF11 in the normal tissue, IGSF11 is highly expressed in gastrointestinal tumors, including colorectal cancer, hepatocellular carcinoma and gastric cancer [32], however, the role of IGSF11 in esophageal carcinoma (which is also a part of digestive tract), is still unknown. In the breast tumor model, the expression of IGSF11 is associated with TGF-β; TGF-β regulates the EMT triggers, which promote the expression of LincRNA Platr18, then induces the expression of IGSF11, and the whole process may be related to the metastasis of breast cancer [33].

**Table 1:** IGSF11/VISTA expression and expression-related regulation mechanisms

	Condition	Expression and Co-expression	Expression Regulation Mechanisms	PMID
IGSF11	Breast cancer		TGF-β regulates the EMT triggers and then promotes the expression of LincRNA Platr18, finally induces the expression of IGSF11	34,810,279
VISTA	Myeloid cells	Raised in intracellular compartment and cell surface in myeloid cells to exert in cell signals exchange;		28,031,817
	Naive T cells	High expression of VISTA in naive T cells may be related to immune tolerance		31,949,051
	Gastric cancer	The expression of VISTA is associated with PD-L1, the co-expression of VISTA and PD-L1 may help gastric cancer patients benefit from combination therapy	The expression of VISTA is associated with promoter methylation, especially in the specific CpG sites; the overexpression of miR-125a-5p can significantly inhibit VISTA expression	28,507,801
	Melanoma	The expression of VISTA is highly associated with the expression of PD-1 and CD33	BRAF and FOXD3 co-participate in the expression of VISTA	32,873,829
	Prostate cancer	After the treatment with ipilimumab, the expression of VISTA is raised higher on CD68 + macrophages, CD4 + T cells and CD8 + T cells		28,346,412
	Breast cancer	VISTA is highly expressed in cytoplasm and membrane and is positively associated with the expression of PD-1, pathological grade and lymph node status		33,250,890
	NSCLC	VISTA is highly accumulated in stromal cell cytoplasm and membrane, and the high level is positively related with the PD-1/PD-L1 axis, and negatively associated with tumor EGFR mutations		30,746,169
		High expression of VISTA are also positively related with CD68 + macrophages and CD8 + T cells, as well as the low mutation burden		29,203,588
	Cervical cancer	VISTA is found expressed in the membrane and cytoplasm of tumor cells, VISTA may co-express with Foxp3, Foxp3 is expressed in CIN I-III, but VISTA only expresses in CIN II-III		34,650,712
	Endometrial cancers	High expression of VISTA is proven on the membrane and in the cytoplasm, especially in G1/G2/G3 histopathologic grades and serous subtypes, associated with high infiltration of CD8 + T cells	The expression of VISTA is modulated by DNA methylation, and the VISTA promoter region 2 may be responsible for the methylation regulation	30,382,166
				34,493,823
	Colon cancer	VISTA is highly associated with CD11b		24,894,088
	Colonrectal cancer	Positively associated with other immune checkpoints like PD1/PD-L1, TIGIT, BTLA and HAVGR2, and positively related with the anti-inflammation factors like Foxp3 and TGFb1, but negatively associated with the Kras mutation		30,128,738
	Pancreatic cancer	Co-express with other immune checkpoints TIM3 and IDO		34,072,549
	Transcription factors		JunD, NF-κB (nuclear factor kappa B) and Fos, bind to the promoter of VISTA and regulate its expression	31,949,051
				22,955,616
	Post-transcriptional regulation		miRNA-125a/miRNA-125b both exert in VISTA expression post-transcriptional regulation by binding VISTA mRNA and inducing its degradation	23,807,506
				22,751,012

VISTA is found highly expressed in normal cells and malignant cells, and exerts in tumor immune regulation. VISTA is raised in tumor infiltration related cells, including T cells, especially in Tregs naïve CD4^+^ and CD8^+^ TCRαβ T cells, and TCRγδ T cells. MDSCs (Myeloid-derived suppressor cells) and macrophages are also found with high expression of VISTA [34–36]. Similar to CTLA-4, VISTA is detected raised in intracellular compartment and cell surface in myeloid cells, which may exert in cell signals exchange [37]. Specifically, VISTA inhibits the activation of TLR-induced IKK/NF-kB and MAPK/AP-1 signal pathways, to regulate the immunosuppression and inflammation of myeloid cells [38, 39]. The high expression of VISTA in naive T cells may be related to immune tolerance [40]. Exclusion for immune cells, VISTA expresses higher in tumors and may interact with IGSF11 in immune regulation, including NSCLC [41], ovarian cancer [42], gastric cancer [43], colorectal cancer [44], soft tissue sarcoma [25] and oral squamous cell carcinoma [45]. High expression of VISTA is found in gastric cancer, especially in EBVa gastric cancer and cancer with liver metastasis, the expression of VISTA is associated with PD-L1, the co-expression of VISTA and PD-L1 may help gastric cancer patients benefit from combination therapy [43]. In melanoma, it has been found that the expression of VISTA is highly associated with the expression of PD-1 and CD33 (MDSCc marker), indicating that both of them may work together in tumor immune inhibition [46]. After the treatment with ipilimumab, the expression of VISTA is raised higher on CD68^+^ macrophages, CD4^+^ T cells and CD8^+^ T cells, given that VISTA is the inhibitory immune checkpoint in prostate cancer, no matter whether for metastatic or localized. This may be the target to explain the ipilimumab treatment resistance and may provide a novel therapy strategy for prostate cancer [47]. In various tumors like breast cancer, NSCLC and gynecological oncology [48–50], VISTA is proven to be expressed on the membrane and in the cytoplasm. Single cell sequencing proves the high expression of VISTA in breast cancer, and the immunohistochemistry indicates that VISTA is highly expressed in cytoplasm and membrane and is positively associated with the expression of PD-1 (*P* = 0.038), pathological grade (*P* = 0.001) and lymph node status (*P* = 0.045) [51]. In NSCLC, VISTA is highly accumulated in stromal cell cytoplasm and membrane, and the high level is positively related with the PD-1/PD-L1 axis, and negatively associated with tumor EGFR mutations [52]. Similar results are proven in 758 NSCLC samples, where high expression of VISTA are also positively related with CD68^+^ macrophages and CD8^+^T cells, as well as the low mutation burden [53]. In cervical cancer, VISTA is found expressed in the membrane and cytoplasm of tumor cells, VISTA may co-express with Foxp3, Foxp3 is expressed in CIN I-III, but VISTA only expresses in CIN II-III, the co-expression of which indicates the prognosis of cervical patients (see part 5.2) [54]. Similarly, high expression of VISTA is proven on the membrane and in the cytoplasm of endometrial cancers, especially in G1/G2/G3 histopathologic grades and serous subtypes, in endometrial cancers, high infiltration of CD8^+^T cells is associated with more VISTA expression. Most subtypes of ovarian cancers like mucinous, serous, clear cell, endometrioid, and undifferentiated carcinoma, all proved to have high VISTA expression, especially in stage I and II cancers [55, 56]. In soft tissue sarcoma, the results of IHC shows that VISTA is highly accumulated on the membrane and cytoplasm of tumor cells, and positively associated with the expression of PD1 and PD-L1 [25], higher expression of PD1 is proved within sarcoma cells [57] and higher PD-L1 membrane expression is mainly found on tumor-infiltrating myeloid cells [58], especially on macrophages, these three immune checkpoints are involved in the regulation of tumor immunosuppression. The expression of VISTA is highly associated with CD11b, in colon cancer, CD11b^+^ cells always have a high VISTA expression, and VISTA^+^ cells have high CD11b expression in lung cancer cells [59], which may be related to antigen presentation and T cell activation. VISTA is found to co-express with other immune checkpoints TIM3 and IDO but is not correlated with survival in pancreatic ductal adenocarcinoma. This is a kind of tumor featured with immune escape, and the immune checkpoints co-expression may be responsible for the immune escape [60]. Similar results are proven in colorectal cancer, the high expression of C10orf54 (encoding VISTA) is positively associated with other immune checkpoints like PD1/PD-L1, TIGIT, BTLA and HAVGR2, and positively related with the anti-inflammation factors like Foxp3 and TGFb1, but negatively associated with the Kras mutation, also meaning that VISTA may be involved in immune escape, thus further investigations are warranted [44].

The expression of VISTA may be modulated by multiple factors, but nearly no reports for IGSF11, the mechanisms to regulate the expression of IGSF11 and VISTA can be found in Table 1. VISTA has its own unique promoter region. There are three potential transcription factors, including JunD, NF-κB (nuclear factor kappa B) and Fos, which bind to the promoter of VISTA and regulate its expression [40, 61]. There are not any known enhancers regulating VISTA expression that have been reported [62]. Besides, miRNA-125a/miRNA-125b both exert in VISTA expression post-transcriptional regulation by binding VISTA mRNA and inducing its degradation [63, 64]. In endometrial cancer, the expression of VISTA is modulated by DNA methylation, and the VISTA promoter region 2 may be responsible for the methylation regulation [55]. The expression of VISTA can be modulated by multiple factors in gastric cancer, and an EMT/MET ( epithelial-mesenchymal transition/ mesenchymal-epithelial transition) model is induced by TGFβ1. In this model, the expression of VISTA is associated with promoter methylation, especially in the specific CpG sites, and VISTA is proven to regulate its downstream effectors, ID2/ID3; besides, the overexpression of miR-125a-5p can significantly inhibit VISTA expression [65]. In melanoma, the inhibition of BRAF promotes the upregulation of FOXD3 (factor Forkhead box D3) by blocking MEK/ERK. FOXD3 serves as the transcription factors of VISTA in melanoma, and the upregulation of FOXD3 can effectively inhibit the expression of VISTA; thus, BRAF and FOXD3 co-participate in the expression of VISTA [66]. Besides, in various cancer cells, keratinocytes and T cells (including MCF-7, Jurkat T, HaCaT keratinocytes, THP-1, K562 and WT-3ab), the expression of VISTA is modulated by TGF-β/Smad3 signal pathway [67], however, the downstream of both of the axes needs further research.

## The interaction between IGSF11 and VISTA

IGSF11 is the specific ligand of VISTA [13]. Co-IP proves the interaction between IGSF11 and VISTA, both SPR (surface plasmon resonance) and FACS (fluorescence-activated cell sorting) assays also prove the specificity between IGSF11 and VISTA [68]. They also prove that after IGSF11 binding with VISTA in V-type and C-type immunoglobulin-like domain, T cell proliferation and related cytokine production can all be inhibited, including IL-17(interleukin-17), CCL3(chemokine ligand 3), CXCL11(C-X-C motif chemokine 11) and CCL5(chemokine ligand 5) [13], and, this provides a theoretical basis for the application of IGSF11 in oncology. IGSF11 antibody and VISTA antibody can effectively block their interaction [27]. SG7 is an antibody targeting VISTA, and there are four epitopes inhibiting VISTA: H122, K38, E125, F36, which overlap with the other two VISTA antibodies, BMS767 and VSTB112 (Bristol Myers Squibb). SG7 can simultaneously compete with the two antibodies and reactivate the function of T cells [69]. The interaction between IGSF11 and VISTA can be affected by the pH of TME (tumor microenvironment), the binding affinity between IGSF11 and VISTA at pH 7.4 is 20 nM, but 80 nM at pH 6.0 (often seen in TME) [69]. HMBD-002 is another kind of antibody targeting the CC’ loop of VISTA, which can effectively block the interaction between VISTA and IGSF11, and further inhibit the production of IFN-γ from IGSF11-mediated T cells. The affinity between HMBD-002 and VISTA can also be affected by the pH, which has the highest affinity at pH 5.5–7.5 [70].

## The biological function of IGSF11 and VISTA in tumors

### The role of IGSF11 and VISTA in immune regulation, mainly in tumors

**Fig. 2:**
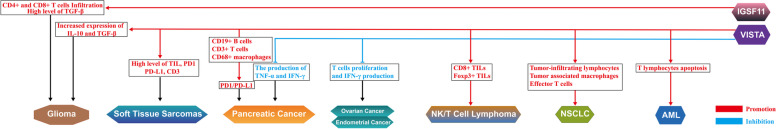
[The immune regulation of IGSF11 and VISTA in tumors]. IGSF11 and VISTA create an inhibitory immune microenvironment in various tumors by affect the function of immune cells and the production of cytokines

Both IGSF11 and VISTA are active in immune cell function and affect cytokine production (Fig. 2). In advanced human gliomas, high expression of IGSF11 is associated with deep immune infiltration, especially for CD4^+^ and CD8^+^ T cells, with high level of TGF-β (*P* < 0.0001), indicating that IGSF11 induces the infiltration of immune cells but weakens their function, finally creating an inhibitory immune microenvironment [71]. Similarly, VISTA is also raised high in glioma, and the expression of IL-10 and TGF-β increases with the increase in VISTA expression [72].

VISTA affects the function of a variety of immune cells, mainly for T cells. VISTA exerts negative immune regulation mainly by inhibiting the activation of T cell receptors, suppressing the proliferation and cytokine production of T cells, but less by affecting cell apoptosis [73, 74]. VISTA may serve with PD1/PD-L1 in immune regulation in tumors, especially for T cell function and activation [59, 75]. In soft tissue sarcomas, high expression of VISTA is found associated with higher level of TIL (tumour-infiltrating lymphocyte, *P* = 0.0033), PD1 (*P* = 0.0046), PD-L1 (*P* = 0.0031) and CD3 (*P* = 0.023). It has been proven that PD1/PD-L1 exert in tumor immune inhibition, which may form a balance with VISTA in soft tissue sarcoma immune regulation [25]. In pancreatic cancer, VISTA may induce the immune deficiency microenvironment, and compared to a melanoma, which is sensitive to immunotherapy, the expression of VISTA is higher on CD68^+^ macrophages in pancreatic cancer. VISTA may significantly inhibit the production of cytokines like TNF-α and IFN-γ; it can also cooperate with PD-L1 in CD8^+^ T-cell inhibition, and the co-inhibition of PD1/PD-L1 and VISTA may help restore the function of T cells in pancreatic cancer [76]. Contrarily, Hou et al. prove that VISTA is highly raised in both tumor cells and immune cells in pancreatic cancer, and especially, that a high level of VISTA is positively associated with CD19^+^ B cells, CD3^+^ T cells and CD68^+^ macrophages [77]. However, further cytological tests are needed to confirm whether the function of these cells is affected. Further, 13F3 (anti-VISTA monotherapy) can effectively improve the tumor microenvironment, reduce the number of tumor specific Treg cells and MDSCs, increase the number of TILs, and promote the function of T cells in melanoma and bladder carcinoma mouse models [34, 78]. In ovarian and endometrial cancer, VISTA can significantly inhibit T cell proliferation and cytokine IFN-γ production, especially for tumor infiltration CD8^+^ T cells, and it also proves that the downregulation of VISTA in endometrial cancer cells can restore T cell proliferation and cytokine production [42, 55]. Naïve mice generate the protective antitumor immunity after being vaccinated with irradiated MCA105 tumor cells, and MCA105 tumor cells are transfected by VISTA-RFP or Control-RFP to express higher VISTA. It proves that higher expression of VISTA can significantly interfere with the protective antitumor immunity, leading tumors to grow vigorously [79]. In NK/T cell lymphoma, the count of CD8^+^ TILs increases with the high expression of VISTA and higher with the co-expression of VISTA and PD-L1, and the single marker high expression of VISTA is correlated with the increase of Foxp3^+^ TILs, but the immune regulation mechanisms are still unknown, besides, high expression of VISTA predicts poor prognosis, thus, maybe the overexpression of VISTA promotes the accumulation of immune cells but inhibits their function? Further exploration is warranted [80]. In NSCLC, high expression of VISTA plays an immunomodulatory function and increases the count of tumor-infiltrating lymphocytes, tumor associated macrophages, effector T cells and PD-1 axis markers [52]. In AML, galectin-9 may be the novel ligand of VISTA and involved in the programmed death of T cells. After the binding between VISTA and galectin-9, caspase-3 and apoptotic death (granzyme B-dependent) are activated, the block of granzyme B can inhibit the apoptotic process, indicating that granzyme B is involved in the apoptosis of T lymphocytes and the interaction between VISTA and galectin-9, as well as the immune regulation of T cells [81]. It is reported that there is no any expression of VISTA in B cells, high expression in naïve CD4^+^ and Foxp3^+^ Regulatory T cells, highest in myeloid cells [17], thus, the immune regulatory role of VISTA for immune cells in tumors and the immune cells subsets in some other immune-related diseases are listed in Table 2.

**Table 2:** The correlations between VISTA and immune cells

Condition	Immune cells	Correlations with VISTA	PMID
Soft tissue sarcomas	TIL	High expression of VISTA is associated with higher level of TIL	35,205,752
Pancreatic cancer	CD68 + macrophages	High expression of VISTA on CD68 + macrophages	30,635,425
	CD8 + T cells	Inhibit the production of TNF-α and IFN-γ	
		Inhibit the function of CD8 + T cells	
Pancreatic cancer	CD19 + B cells	high level of VISTA is positively associated with CD19 + B cells, CD3 + T cells and CD68 + macrophages	33,237,432
	CD3 + T cells		
	CD68 + macrophages		
Melanoma	Treg	The block of VISTA reduces the number of tumor specific Foxp3 + CD4 + Treg cells, the presence of activated dendritic cells and MDSCs, increases the number of TILs, and promotes the function of T cells	24,691,994
	MDSCs		
	DCs		
	TIL		
Ovarian and endometrial cancer	CD8 + T cells	VISTA can significantly inhibit T cell proliferation and cytokine IFN-γ production	30,127,950
			30,382,166
NK/T cell lymphoma	CD8 + TILs	High expression of VISTA is correlated with the increase of CD8 + TILs and Foxp3 + TILs	33,889,438
	Foxp3 + TILs		
NSCLC	TIL	High expression of VISTA increases the count of tumor-infiltrating lymphocytes, tumor associated macrophages, effector T cells	30,746,169
	Tumor associated macrophages		
	Effector T cells		
Melanoma	MDSCs	VISTA is expressed on CD33 + myeloid cells and positively associated with the expression of CD33	32,873,829
VISTA-mediated anti-tumor immunity	Macrophages	The absence of VISTA promotes the production of proinflammatory cytokines of TLR-mediated in peritoneal macrophages	31,340,983
	MDSCs subsets		
	DCs subsets	VISTA regulates the effector function and accumulation of tumor-associated myeloid cDCs, inflammatory DCs, CD103 + DCs, PMN-MDSCs and M-MDSCs	
Allergic asthma	Th2	VISTA involves in the generation of Th2 cells and related antibody production	29,267,882
Maintaining naïve T cell quiescence and tolerance	Naïve CD4 + T cells	The absence of VISTA enhance the response of CD44hi CD4 + memory-like T cells to TCR and cytokine stimulation	31,949,051
	Antigen-specific T cells	The block of VISTA induces the tolerance reduction of antigen-specific T cells	
Giant cell arteritis	Th1	The block of VISTA promotes the differentiation of Th1 and Th17 cells	31,379,838
	Th17		
Anti-VISTA mAbs research	Monocytes	The administration of anti-VISTA mAb induces the activation of monocytes	34,106,206
Collagen antibody-induced arthritis	Macrophages	VISTA regulates the response of macrophages to immune complexes	29,216,931
Glomerulonephritis	Neutrophil	The block of VISTA inhibits the activation of neutrophil	32,586,651

### The role of IGSF11 and VISTA in tumor growth, proliferation

**Fig. 3:**
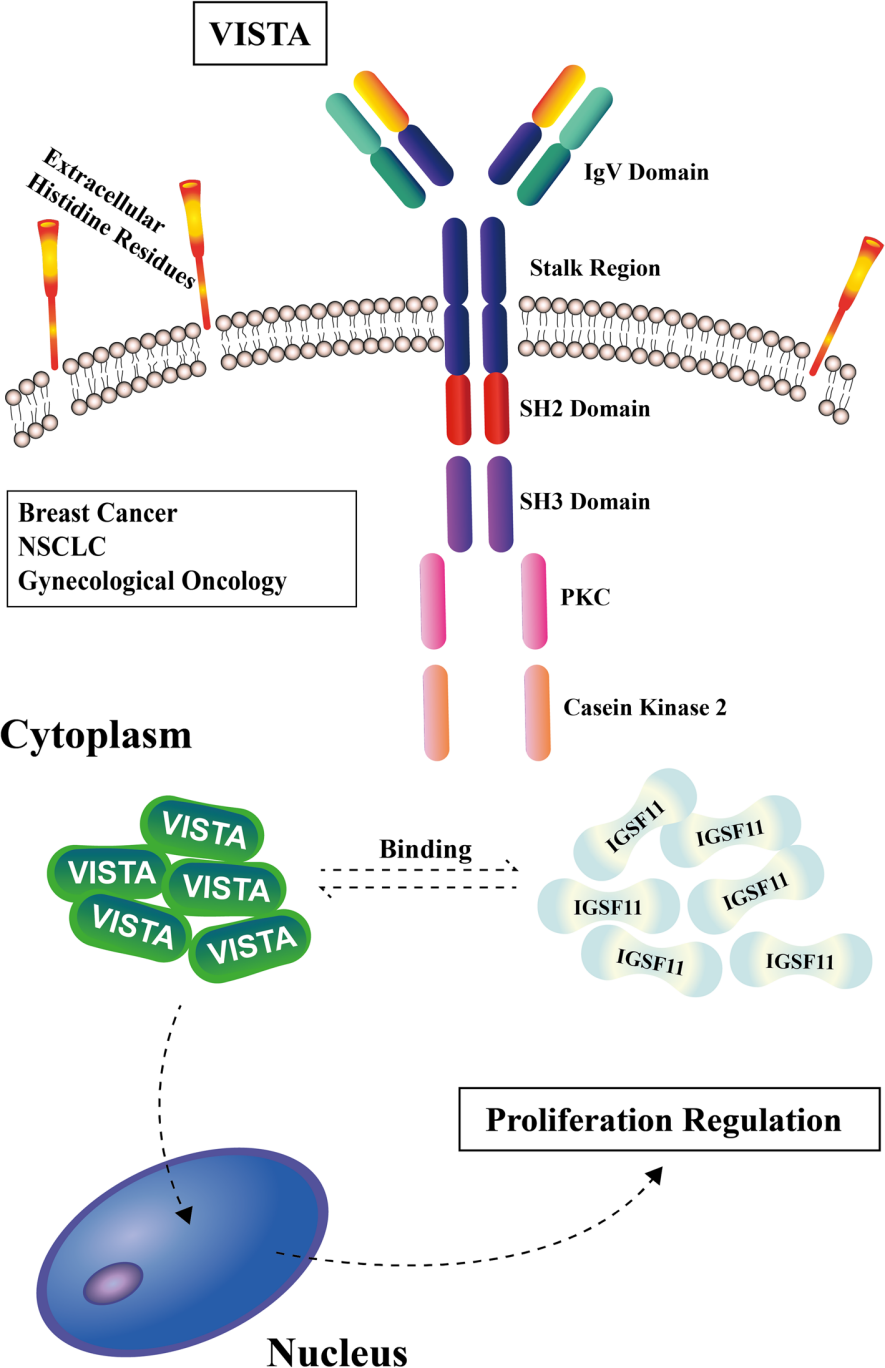
[The possible proliferation regulation of IGSF11 and VISTA in the cytoplasm of tumor cells]. VISTA is proved highly accumulated in the cytoplasm of above tumors, may exert its proliferative role with IGSF11

Both IGSF11 and VISTA may be involved in tumor growth. In gastric cancer St-4 cells, the reduced expression of IGSF11 decreases the transfected St-4 cells number, the growth inhibitory effect can also be found in NIH3T3 cells and proved by colony formation assay in gastric cancer, but the intrinsic mechanisms are still unclear [32]. Besides, Katoh et al. indicate that IGSF11 is associated with some adhesion molecules’ encoding, like ESAM, FLJ22415 and CXADR, which may involve cell adhesion and drug delivery in gastric cancer [7]. In the fibrosarcoma mouse model, the overexpression of VISTA may induce the inhibition of T cells and boost the growth of tumor cells [79]. Similar results can be found in the glioma mouse model; VISTA-deficiency mice have a slower tumor growth rate, therefore, the block of VISTA may serve in tumor growth inhibition [82]. The melanoma animal model shows that the block of VISTA promotes the proliferation, infiltration and effector role of T cells, reducing the count and inhibiting the activation of MDSCs and Tregs, finally, boosting the growth of the melanoma [34]. In PDAC (pancreatic ductal adenocarcinoma), the expression of VISTA may be associated with TLR4; the downregulation of VISTA and TLR4 by siRNA and naloxone, respectively, can inhibit the growth of PDAC, indicating that VISTA and TLR4, with their downstream signal pathways, are all involved in PDAC growth [83]. Given that VISTA is also accumulated in cytoplasm of tumor cells, we consider that VISTA may bind with IGSF11 in cytoplasm and exert in tumor proliferation, but this hypothesis warrants more confirmation (Fig. 3).

## The preclinical and clinical research progression of IGSF11 and VISTA

### The research about targeted drugs

Both IGSF11 and VISTA have the potential to be the novel targets in tumor immunotherapy (Table 3). In gastric cancer, based on IGSF11, the polypeptide vaccine that is designed can effectively enhance the function of CTLs (cytotoxic T lymphocytes), which are identified for their favorable role in tumor inhibition and patients’ survival [32]. SG7 is such an antibody inhibiting the interaction between IGSF11 and VISTA. In the melanoma mouse model, the administration of SG7 for two weeks at 10 mg/kg can effectively inhibit the tumor growth; further, SG7 combined with anti-PD1 in the MC38 colon carcinoma model, also obtained similar results, which is a good sign for clinical application [69].

**Table 3:** IGSF11 and VISTA targeted drugs

Tumor	Target	Drug	Mechanisms	PMID
Gastric cancer	IGSF11	Polypeptide Vaccine	Enhance the Function of CTLs	16,108,831
Melanoma	IGSF11	SG7	Inhibit the interaction between VISTA and IGSF11	32,938,950
Colon Carcinoma	IGSF11/PD1	SG7 combined with anti-PD1	Inhibit the interaction between VISTA and IGSF11; anti-PD1	32,938,950
Colon Carcinoma	VISTA/PD-L1	VISTA-Ig and PD-L1 Ig	anti-VISTA and anti-PD-L1	25,964,334
			Enhance the response of T cells	
			Increase the count of T cells	
			Boost antigen presentation	
Advanced Solid Tumors or Lymphomas	VISTA/KRAS	CA170 and KRAS peptide vaccine	Boost the infiltration of CD8 + T cells	34,302,042
			Decrease the infiltration of MDSCs	
			Decrease the infiltration of Tregs	
			Inhibit lung tumorigenesis	
Solid tumor	VISTA	CI-8993	anti-VISTA	33,937,071
Melanoma	VISTA	13F3	Inhibit the proliferation of tumor	24,691,994
			Induce the apoptosis of tumor cells	
			Increase the count of IFN-γ producing cells	
			Promote the response of tumor specific T cells	
Melanoma	VISTA	αVISTA	Increase the count of CD4 + and CD8 + T cells	24,691,994
			Decrease the infiltration of MDSCs	
Bladder tumor	VISTA	13F3	Activate tumor-associated CD11c + DCs	24,691,994
			Induce the production of IL-12 and TNF-α	
			Decrease the count of Foxp3GFP + iTregs	
			Directly inhibit Tregs activation	
			Enhance the proliferation and cytotoxic function of CD8 + T cells	
Mouse model	VISTA	6809–0223	Promote the proliferation of CD4 + T cell	34,380,434
			Induce the production of IL-2 from both CD4 + and CD8 + T cell	
			Induce the production of IL-4, IFN-γ and TNF-α from CD8 + T cell	
Colon cancer	VISTA	HMBD-002	Promote the response of proinflammatory Th1 cells	35,131,861
Breast cancer			Increase the number of CD11b + macrophages, CD11c + DCs and CD8 + T cells	
Colorectal cancer				
Lung cancer				
Squamous cell carcinoma	VISTA/CTLA4	MIH63	Activate and upregulate CD8 + T cells	34,282,763
			Converse the exhausted cells into effector CD8 + T cells	27,208,845
			Promote the secretion of TNF- α and IFN-γ	

There are only 3 clinical trials targeting VISTA: JNJ-61610588, CA-170 and CI-8993 (NCT02671955, NCT02812875, NCT04475523). JNJ-61610588 is the first anti-VISTA antibody in clinical trial for solid tumors, but is terminated at present [84], but the studies about CA-170 get progress rapidly. The combination therapy of CA170 and KRAS peptide vaccine can effectively inhibit lung tumorigenesis in the mouse model. The administration of CA170 helps in boosting the infiltration of CD8^+^ T cells, decreasing the infiltration of MDSCs and Tregs, and exerting its potent anti-tumor effect, especially combined with the KRAS vaccine [85]. Clinical trial NCT02812875 has been completed and proved the potent anti-tumor effect of CA-170 [86], besides, in this clinical trial which enrolled 59 patients (13 NSCLC, 10 colorectal cancer, 8 SCCHN, 5 ovarian cancer, 4 melanoma, 3 renal cell carcinoma, 2 breast cancer, 2 esophageal cancer, 2 Hodgkin's Lymphoma, 2 Non-Hodgkin's Lymphoma, 8 other tumors), CA170 was administered 1200 mg twice daily in 21 days cycles. Of the patients, 33 patients had the best response based on RECIST (response evaluation criteria in solid tumors), but some reported grade 1–2 irAEs like nausea, constipation and fever, and some experienced grade 3–4 irAEs such as increased blood bilirubin, and amylase increase, which all occurred during the process of treatment [84].

CI-8993 is another antibody targeting VISTA, which is in a phase I clinical trial recruiting 50 relapsed and/or refractory solid tumor patients to evaluate its safety [87]. In the B16OVA melanoma model and MB49 bladder tumor model, VISTA mAbs show significant therapeutic effect. For the B16OVA melanoma model, the performance of 13F3 (VISTA mAb) can effectively inhibit the proliferation of tumor and induce its apoptosis, increase the count of IFN-γ–producing cells and promote the response of tumor specific T cells. Besides, the administration of αVISTA (VISTA mAb) shows similar anti-tumor effect in the B16OVA melanoma model, where the count of CD4^+^ T cells and CD8^+^ T cells increased (6.38% to 11.74%, 9.25% to 17.74%, respectively), and the percentage of MDSCs significantly decreased (37.74% to 25.64). For the MB49 bladder tumor model, the performance of VISTA mAb can effectively inhibit tumor growth by activating tumor-associated CD11c^+^DCs and inducing the production of IL-12 and TNF-α. Furthermore, VISTA expresses higher in CD62L^−^ and ICOS^−^ Tregs, and the block of VISTA decreases the count of Foxp3GFP^+^ iTregs and directly inhibit Tregs activation, enhancing the proliferation and cytotoxic function of CD8^+^ T cells [34].

The compound 6809‑0223 is a small molecule and a hit ligand with an excellent binding rate when binding with VISTA-ECD (extracellular domain). This can significantly promote the proliferation of CD4^+^ T cells, and induce the production of IL-2 from both CD4^+^ and CD8^+^ T cells, and the production of IL-4, IFN-γ and TNF-α from CD8^+^ T cells, thus exerting potent immune regulation in the mouse model. The security and possibility for clinical application warrants further evaluation [88]. HMBD-002 is a kind of antibody specifically binding to the CC’ loop of the antibody and is involved in inhibiting IFN-γ secretion. HMBD-002 remodels the immune microenvironment and promotes the response of proinflammatory Th1 cells, and increases the number of CD11b^+^ macrophages, CD11c^+^ DCs and CD8^+^ T cells. This shows potent anti-tumor effects in the CT26 colon cancer mouse model, 4T1 breast cancer model, HCT15 colorectal cancer model and A549 lung cancer model, where the tumor growth inhibition rates are 84%, 53%, 65% and 62%, respectively [70].

In the CT26 colon carcinoma mouse model, the combination therapy for anti-VISTA and anti-PD-L1 mAbs (VISTA-Ig and PD-L1 Ig) may induce 80% tumor regression by enhancing the response and increasing the count of T cells, boosting the antigen presentation and T cells’ activation [89], which may be attributed to the synergistic effect of VISTA and PD-1 in tumor immune regulation. Compared to the combination therapy of anti-PD-1 with anti-VISTA, the combination between anti-CTLA-4 and anti-VISTA (MIH63) shows stronger anti-tumor effect in the squamous cell carcinoma cell model, significantly slowing the growth of the tumor by activating and upregulating CD8^+^ T cells, converting the exhausted cells into effector CD8^+^T cells, and promoting the secretion of TNF- α and IFN-γ [90, 91].

### Immunotherapy resistance

VISTA may be involved in PD1/PD-L1 treatment resistance. About 50% patients develop the anti- PD-L1 resistance during the treatment and this can be attributed to the problem of antigen presentation and T cell exhaustion [92, 93]. It was also found that VISTA raised higher expression after the treatment of anti-PD1 or anti-CTLA-4 [53]. These two phenomena may be linked together and VISTA may contribute to the resistance immunotherapy, and the combination therapy of anti-VISTA and anti-PD1 may delay the progression of resistance. Clinical trial NCT02812875 aims at evaluating the function of CA170 (anti- VISTA/PD-L1) in solid tumor or lymphoma, which has proved its potent antitumor effect and may help to get a higher immune response rate [86]. VISTA may also be involved in PD-1 resistance in NK/T cell lymphoma. Patients with low expression of VISTA show no response to anti-PD-1 therapy but those with high expression show complete remission, which can even be maintained for more than 12 months [80]. VISTA may also induce the anti-PD-1 resistance in metastatic melanoma. Kakavand et al. found that from pretreatment to disease progression, the expression of VISTA (*P* = 0.009), PD-L1 and FOXP3 all increased during the process. Given the negative immune regulation function of VISTA in tumors, VSITA may be involved in the failure of anti-PD-1 therapy; however, the mechanisms of VISTA in metastatic melanoma immunotherapy resistance are still unclear, and warrant further exploration [94]. The combination therapy of cyclophosphamide, radiation therapy, plus the dual block of PD-1/VISTA, may exert a potent anti-tumor effect in metastatic triple negative breast cancer. This has been proven in the 4T1 tumor mouse model, and, most importantly, this strategy can effectively improve the anti-PD-1 therapy resistance and inhibit lung metastasis by activating CD8 + T cells and decreasing the counts of MDSCs (53.55% vs. 19.9). Further, the combination of radiation therapy with anti-VISTA can also decrease the number of MDSCs in tumors [95].

### Prognosis prediction

**Fig. 4:**
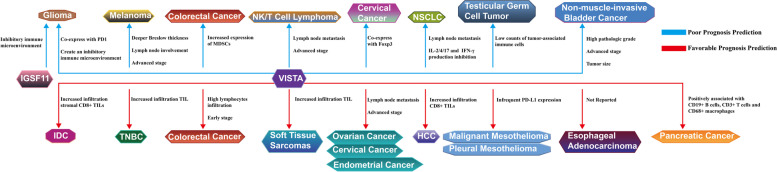
[The prognosis prediction of IGSF11 and VISTA in various tumors]. The top half shows the poor prognosis prediction and possible relevant factors of IGSF11 and VISTA, the bottom half indicates the favorable prognosis prediction and possible relevant factors of VISTA, in various tumors

Both IGSF11 and VISTA can predict the poor prognosis in tumors (Fig. 4). The survival curves with IGSF11 and VISTA for 19 tumors can be found in Supplementary Fig. 1.

At present, the relationship between IGSF11 and tumor prognosis prediction is only reflected in glioma, which may coordinate with PD-1, especially in advanced human gliomas, the high expression of IGSF11 is associated with poor overall survival (*P* = 0.0004) the high co-expression of PD-1 and VISTA in advanced human gliomas show poorer survival (*P* < 0.0001, *P* = 0.0078), the protein level of IGSF11 is not related to tumor grades and histologic type, but IGSF11 is found expressed in tumor samples of all grades, on both glioma cells and tumor-associated inflammatory cells. indicating that IGSF11 may serve as a ligand and receptor simultaneously, to interact with PD-1 and VISTA to create an inhibitory immune microenvironment, which is related to the poor prognosis [71], however, this study only proved the role of IGSF11 in advanced glioma, but the prognostic role of IGSF11 in other tumors has not been demonstrated, and there is a great space for scientific research and exploration. VISTA also predicts the poor prognosis of glioma patients (*P* = 0.0085); VISTA may co-express with PD1, and high expression of both the immune checkpoints indicate worse survival (*P* < 0.0001), which is associated with the negative regulation of VISTA in the glioma microenvironment [72].

High expression of VISTA predicts the poor prognosis in following tumors, which is associated with tumor immune microenvironment. In melanoma the co-expression of VISTA and CD33 (MDSC marker) predicts a worse prognosis,the high expression of VISTA is associated with the occurrence of ulceration (*P* = 0.015), deeper Breslow thickness (*P* = 0.002), lymph node involvement (*P* < 0.001) and advanced stage (*P* = 0.008), specially, in 30% cutaneous melanoma patients, co-expression of VISTA and PD-1 co-mediate immunosuppression, further it proves that the expression of PD-1 can be affected by CD33^+^ MDSCs, all above indicate that VISTA, PD-1 and MDSCs may co-participate in melanoma immunosuppression and further induce poor prognosis of patients [46]. In NK/T cell lymphoma, high expression of VISTA is correlated with more infiltration of Foxp3^+^ Tregs, both expressions of VISTA (*P* = 0.001, HR = 2.05, 95% CI: 1.29–3.25) and PD-L1 (*P* = 0.005, HR = 1.93, 95% CI: 1.22–3.07), respectively, are risk factors and predict poor PFS (progression free survival) and OS (overall survival), the upregulation of VISTA and PD-L1 inhibit the activation and proliferation of CD8^+^T cells, which are associated with lymph node metastasis (*P* = 0.004) and the advanced stage (*P* = 0.002) [80]. In lung adenocarcinoma, the high expression of VISTA in CD4^+^ T cells is associated with a reduction in the overall survival (*P* = 0.03), lymph node metastasis (*P* = 0.05) and cytokine production inhibition, including IL2/4/10/17, and IFN-γ, the inhibitory tumor immune microenvironment may be associated with the poor prognosis of patients, moreover, the increased infiltration of CD4^+^ VISTA^+^ T cells is associated with advanced pathology Node (pN) staging but not for Tumor (pT) staging, however, the role of VISTA in lung squamous carcinoma or other types is still unclear [96]. In testicular germ cell tumors, the low counts of tumor-associated immune cells expressed with PD-L1 and VISTA may be related to stage I patients’ relapse but not for stage II and stage III, the high platelet-to-lymphocyte ratio and low counts of VISTA express tumor-associated immune cells which are the biomarkers to predict worse prognosis of testicular germ cell tumors in patients (HR = 15.56, *P* = 0.001 and HR = 4.1, *P* = 0.006, respectively) [97]. In non‑muscle‑invasive bladder cancer, the positive expression of VISTA may indicate the short recurrence of bladder cancer with recurrence-free survival, compared to the negative ones (34.0 vs. 39.9 months, *P* = 0.03). Moreover, high expression of VISTA is associated with advanced tumor stage (67.7% vs. 32.3%, pT1 vs. pTa and pTis, *R* = 0.325, *P* < 0.001), high pathologic grade (71.0% vs. 29.0%, pT1 vs. pTa and pTis, *R* = 0.438, *P* < 0.001) and tumor size larger than 3 cm (*R* = 0.322, *P* < 0.001) [98].

In some tumors, high expression of VISTA predicts a favorable prognosis with more infiltration of TILs. In soft tissue sarcomas, high expression of VISTA may predict the favorable prognosis of patients (*P* = 0.043), which can be attributed to the increased TIL, especially for CD3^+^ cells, however, more frequent VISTA is found in higher FNCLCC grade (G3 vs. G2, *P* = 0.019), exactly opposite of what is predicted without further explanation [25]. In TNBC (triple-negative breast cancer), Cao et al. indicate that higher expression of VISTA is less associated with lymph node metastasis but more related to the infiltration of TILs, especially for CD4^+^ TILs, more VISTA^+^ immune cells are found in stage I/ II patients and basal-like subtype with favorable OS, especially for T1-2N0 stage patients [99]. Similar results can be found in IDC (invasive ductal carcinoma) of the breast, proving that VISTA is accumulated more in the subtypes of EGFR2^+^, and positively associated with the expression of PD1/PD-L1, as well as stromal CD8^+^ TILs (*P* < 0.001), predicting the favorable disease-specific survival and relapse free survival of IDC patients, especially in basal-like, ER^−^ and PR^−^ IDC subtypes, however, high VISTA expression is also found in EGFR2^−^, EGFR2^+^ and poorly differentiated subtypes, but not related with prognosis prediction [100]. In all types of malignant mesothelioma, the survival analysis shows that VISTA is an independent favorable prognostic factor (*P* = 0.008); these results are confirmed by a multivariate survival analysis (*P* = 0.014, 95% CI = 1.25–7.72) [101], especially, in pleural mesothelioma, VISTA expression predicts the favorable prognosis but PD-L1 expression predicts the worse (*P* = 0.001, *P* = 0.002, respectively), but the expression of PD-L1 is infrequent, which may induce the insensitivity of pleural mesothelioma patients to anti-PD-L1 treatment [102]. In pancreatic cancer, high expression of VISTA is found in tumor cells, positively associated with the infiltration of CD19^+^ B cells, CD3^+^ T cells and CD68^+^ macrophages, besides, frequent VISTA expression is a risk factor of tumor grade (HR = 3.911, *P* = 0.012), T stage (HR = 2.885, *P* = 0.006), N stage HR = 4.221, *P* = 0.001) and M stage (HR = 5.57, *P* = 0.001) but a protective factor for patients survival ( HR = 0.588, *P* = 0.029) [77]. VISTA improves the prognosis of esophageal adenocarcinoma, especially for T1/T2 tumors with VISTA^+^ TILs, compared to the tumors without VISTA expression (median overall survival is 21.6 months, 95%CI 13.3–29.9 months), the tumors with high VISTA expression have better prognosis (median overall survival is 202.2 months, 95%CI 32.6–371.8 months), however, it has proved that this benefit is not seen in advanced esophageal adenocarcinoma stages and unclear in other pathological subtypes of esophageal neoplasms [103]. In HCC (Hepatocellular carcinoma), the expression of VISTA is associated with CD8^+^ TILs (*P* < 0.001) and higher pathological grades (III-IV) but not related with TNM stages, and the double positive expression of VISTA and CD8 has the most favorable prognosis of HCC [104].

The prognostic role of VISTA is contradictory in colorectal cancer and cervical cancer. In colorectal cancer, hypoxia can induce HIF1α (hypoxia-inducible factor 1-alpha), binding to the promoter of VISTA to increase the expression on MDSCs, enhance the inhibition of MDSCs to T cell function, which can be the reason for high VISTA expression indicating the poor prognosis of colorectal patients, however, the survival analysis is on the foundation of GSE40967, the survival association is confirmed only at the mRNA level but not in protein level [105]. Besides, compared to the patients in the early stage, the mRNA level of VISTA in circulation is significantly upregulated in the advanced stage, which may indicate the poor prognosis of colorectal cancer patients [106]. However, Wu et al. consider that the high expression of VISTA predicts the better prognosis of colorectal cancer patients (*P* = 0.005), with high lymphocyte infiltration and low tumor stage, high AJCC III-IV stages tumors have lower VISTA expression, the order of VISTA expression is as follows: pT1 > pT2 > pT3 > pT4 [107]. Zong et al. also provide similar results for survival analysis [108]. In a word, the differences in VISTA prediction of colorectal cancer survival can be attributed as follows, the first two studies only complete the survival analysis of VISTA mRNA level but not for protein level, besides, the cutoff of VISTA expression may decide optimized, the median cutoff is not correlated with tumor survival in GSE40967 but optimized cutoff does [105].

The double positive expression of VISTA and Foxp3 indicate the worst prognosis of cervical cancer patients, compared to the single negative group; the survival analysis shows that the single positive expression of VISTA has the worst average survival (35.114 ± 2.828 vs. 51.486 ± 1.403 months). The 3-year survival rate (54.3% vs. 91.4%), similarly, compared to the double negative group, the double positive expression of VISTA and Foxp3 shows the worst average survival (26.813 ± 3.584 vs. 52.929 ± 1.052), especially for patients in higher stage because VISTA and Foxp3 both can be found higher in stage II cervical cancer [54]. In ovarian, cervical, and endometrial cancer, high expression of VISTA is related to the advanced stage (stage II, III, IV) and lymph node metastasis, and positively associated with prolonged survival [42, 109]. Especially, in high‑grade serous ovarian cancer, the expression of the VISTA encoding gene C10orf54 also indicates the prolonged overall survival (*P* = 0.004) [49]. There is lack of convincing explanation for the differences in the prognosis prediction of VISTA in cervical cancer, which may be attributed to sample heterogeneity.

## Perspective

Immunotherapy has become the major treatment for tumors, but the problem of the low response rate and the high immune-related adverse events remain unsolved. Thus, novel immunotherapy targets warrant further exploration. According to our review and bibliometric study, VISTA and its ligands, especially for IGSF11, may be the novel and promising target in tumor immunotherapy [110, 111].

The crystal structure of both IGSF11 and VISTA have been identified, which may provide more targets for antibody design. The V-type and C-type immunoglobulin-like domain of IGSF11 are responsible for binding with VISTA, SG7 is a kind of antibody that inhibits the interaction between IGSF11 and VISTA. An in-depth analysis of the VISTA structure finds the CC’ loop region of VISTA is also a target for antibody design (HMBD-002). Thus, it is of great clinical significance to further explore the IGSF11 structure, which may provide further rationale for anti-IGSF11 targeted drug design.

IGSF11 proved to be highly accumulated in tumor cells and VISTA raised more in both immune cells and malignant cells; the expression of these are modulated by signal pathways and epigenetic regulation. Considering the differences of IGSF11 and VISTA studies in different tumors, we consider that the role of IGSF11 in non-small-cell lung cancer, ovarian cancer and oral squamous cell carcinoma, even in esophageal carcinoma, worth further exploration. Besides, more research focuses on the expression regulation signal pathways and epigenetic regulation of VISTA but less on IGSF11, and studying the corresponding IGSF11 expression regulation mechanism may provide more targets for IGSF11 downregulation and contribute further to tumor immunotherapy.

At present, the high affinity between IGSF11 and VISTA has been proved, however, after IGSF11 binds to VISTA, the related inhibitory signals occur and are involved in immune inhibition, but the specific intracellular mechanisms and signals are still unknown, and warrant further investigation.

Both IGSF11 and VISTA exert an inhibitory function in immune regulation. IGSF11 and VISTA can both inhibit the function of TILs, especially for CD8^+^ T cells, besides, both promote the production of TGF-β and IL-10, inhibit the production of TNF-α and IFN-γ, and finally exert immunosuppression in tumors. Various studies have proved that VISTA coordinates with PD1/PD-L1 in tumor immune regulation but not for IGSF11 [112]. Even the immune regulation of IGSF11 has just been proved in glioma, but VISTA has been proved to be in many other tumors, thus, the research potential for the mechanisms of IGSF11 in tumor immune regulation is enormous, since the intracellular mechanisms are still unclear. Most studies have found a negative relationship between IGSF11/VISTA and immunity in some tumors, and just exhibit the relationship between gene expression and cell count, but there has been a lack of sufficient cytological trials to prove the positive relationship and thus, this has not been sufficiently convincing.

Both IGSF11 and VISTA may be involved in tumor growth. The downregulation of either IGSF11 or VISTA in various tumor models, significantly inhibits tumor growth, and the mechanisms of VISTA may be attributed to the restoration of T cells, but the mechanisms of IGSF11 are still unknown. We consider that VISTA locates on the membrane and in the cytoplasm of tumors may directly interact with IGSF11 and affect the proliferation of tumors, the discovery of this phenomenon may suggest a new hypothesis: IGSF11 and VISTA exert a double regulation in tumors, which include both immune regulation and proliferation regulation—which is also a novel direction for exploration of the immune checkpoints.

Both IGSF11 and VISTA have the potential to be the novel target in tumor immunotherapy. Currently, three drugs (JNJ-61610588, CA-170 and CI-8993) targeting VISTA are ongoing clinical trials, other VISTA inhibitors like 13F3, αVISTA, 6809‑0223, HMBD-002, MIH63 have tested their therapeutic effect in mouse tumor models, most of them exert by restoring or enhancing the function of immune cells, and promoting the production of cytokines like IL-4, IFN-γ and TNF-α. Compared to VISTA, few studies focus on anti-IGSF11 drugs. SG7 is such a kind of antibody that inhibits the interaction between IGSF11 and VISTA. Further, IGSF11-related peptide drugs have emerged and proved their efficacy in a variety of tumors, but there is still a need to explore and further expand the scope of tumor adaptation, and the corresponding clinical validation is lacking. In addition to monotherapy, combination therapy is also an important research field, and the combination therapy of anti-VISTA/anti-IGSF11 and anti-PD1/PD-L1, may produce better therapeutic effects in tumors [113–115]. Current research has found the high expression of VISTA in anti-PD1/PD-L1 resistance samples, thus, combination therapy may simultaneously improve the clinical immunotherapy resistance and the occurrence of irAEs should be emphasized during the process of combination therapy.

Both IGSF11 and VISTA can predict the prognosis in tumors. IGSF11 predicts the favorable prognosis of tumors but VISTA predicts both the favorable and poor prognosis in various tumors, and the expression of this pair of immune checkpoints is associated with other immune checkpoints and immune markers. VISTA is negatively associated with tumor immune regulation but improves the infiltration of TILs in some tumors like soft tissue sarcomas and TNBC, which can be a possible reason that explains the high level of VISTA’s ability to predict favorable prognosis in these tumors.

Taken together, IGSF11 and VISTA are both highly accumulated in tumors, and participate in the regulation of tumor growth, immune microenvironment, therapy resistance and prognosis prediction. As a whole, more research focuses on VISTA and less on IGSF11, but we consider that IGSF11 also plays a strong regulatory role in a variety of tumors, although the specific role and regulatory mechanism are unclear. The present research has proved the predictive role of IGSF11 and VISTA in tumors, but whether the anti-VISTA/anti-IGSF11 treatment is indeed effective and can improve the prognosis of patients is still unknown. There is a lack of data about patient survival and a contributory factor is due to the delays in the targeted drug studies. This may also be due to the fact that the specific mechanism and site of action of IGSF11 and VISTA in tumors have not been effectively developed, thus, relevant drug research and clinical research are needed for further exploration.

## Supplementary Information

**Additional file 1: SupplementaryFigure 1.** [The survival curvesof IGSF11 and VISTA in 19 tumors]. The survival curves of IGSF11 and VISTA, 19tumors are included: BLCA (Bladder Urothelial Carcinoma), BRCA (Breast invasivecarcinoma), CESC (Cervical squamous cell carcinoma and endocervicaladenocarcinoma), COAD (Colon adenocarcinoma), DLBC (Lymphoid Neoplasm DiffuseLarge B-cell Lymphoma), ESCA (Esophageal carcinoma), GBM (Glioblastomamultiforme), LAML (Acute Myeloid Leukemia), LGG (Brain Lower Grade Glioma),LIHC (Liver hepatocellular carcinoma), LUAD (Lung adenocarcinoma), LUSC (Lungsquamous cell carcinoma), MESO (Mesothelioma), OV (Ovarian serouscystadenocarcinoma), PAAD (Pancreatic adenocarcinoma), SARC (Sarcoma), SKCM(Skin Cutaneous Melanoma), TGCT (Testicular Germ Cell Tumors), UVM (UvealMelanoma).

## Data Availability

The materials that support the conclusion of this review have been included within the article.

## Abbreviations

PD1/PD-L1: Programmed Cell Death 1/ Programmed Cell Death Ligand 1
CTLA4: Cytotoxic T-Lymphocyte Associated Protein 4
LAG3: Lymphocyte Activating 3
irAEs: Immune-related adverse events
IGSF11: Immunoglobulin superfamily 11 gene
VISTA: V-domain immunoglobulin suppressor of T cell activation
PSGL1: P-selectin glycoprotein ligand 1
SH2 domain: Src homology domain 2
SH3 domain: Src homology domain 3
MDSCs: Myeloid-derived suppressor cells
NSCLC: Non-small-cell lung cancer
NF-κB: nuclear factor kappa B
EMT/MET: Epithelial-mesenchymal transition/ mesenchymal-epithelial transition
FOXD3: Factor Forkhead box D3
SPR: Surface plasmon resonance
FACS: Fluorescence-activated cell sorting
IL-17: Interleukin-17
CCL3: Chemokine ligand 3
CXCL11: C-X-C motif chemokine 11
CCL5: Chemokine ligand 5
TME: Tumor microenvironment
TIL: Tumour-infiltrating lymphocyte
PDAC: Pancreatic ductal adenocarcinoma
CTLs: Cytotoxic T lymphocytes
RECIST: Response evaluation criteria in solid tumors
ECD: Extracellular domain
PFS: Progression free survival
TNBC: Triple-negative breast cancer
IDC: Invasive ductal carcinoma
