# Supplementary figures and images for: IGSF11 and VISTA: a pair of promising immune checkpoints in tumor immunotherapy
Source: Biomark Res. 2022 Jul 13;10:49. doi: 10.1186/s40364-022-00394-0 (PMC9277907; doi:10.1186/s40364-022-00394-0)

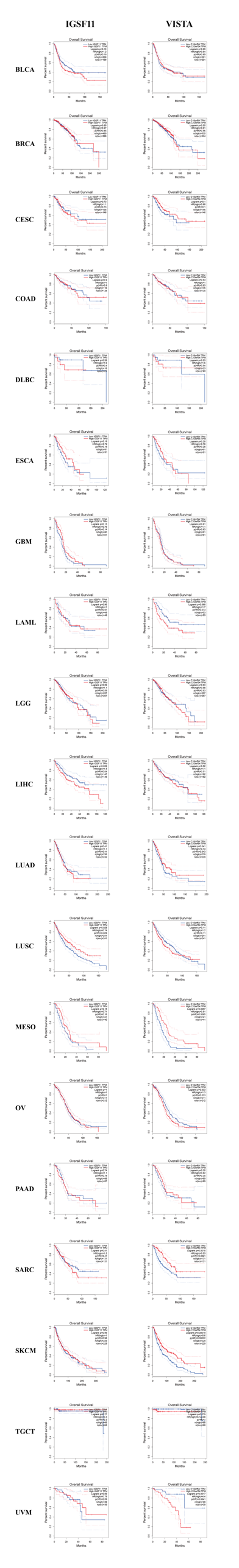

Supplement: Supplementary file 1 — Additional file 1: SupplementaryFigure 1. [The survival curvesof IGSF11 and VISTA in 19 tumors]. The survival curves of IGSF11 and VISTA, 19tumors are included: BLCA (Bladder Urothelial Carcinoma), BRCA (Breast invasivecarcinoma), CESC (Cervical squamous cell carcinoma and endocervicaladenocarcinoma), COAD (Colon adenocarcinoma), DLBC (Lymphoid Neoplasm DiffuseLarge B-cell Lymphoma), ESCA (Esophageal carcinoma), GBM (Glioblastomamultiforme), LAML (Acute Myeloid Leukemia), LGG (Brain Lower Grade Glioma),LIHC (Liver hepatocellular carcinoma), LUAD (Lung adenocarcinoma), LUSC (Lungsquamous cell carcinoma), MESO (Mesothelioma), OV (Ovarian serouscystadenocarcinoma), PAAD (Pancreatic adenocarcinoma), SARC (Sarcoma), SKCM(Skin Cutaneous Melanoma), TGCT (Testicular Germ Cell Tumors), UVM (UvealMelanoma). [file 40364_2022_394_MOESM1_ESM.tif]
